# Semi-Correlations for Building Up a Simulation of Eye Irritation

**DOI:** 10.3390/toxics11120993

**Published:** 2023-12-06

**Authors:** Andrey A. Toropov, Alla P. Toropova, Alessandra Roncaglioni, Emilio Benfenati

**Affiliations:** Istituto di Ricerche Farmacologiche Mario Negri IRCCS, Via Mario Negri 2, 20156 Milano, Italy; andrey.toropov@marionegri.it (A.A.T.); alessandra.roncaglioni@marionegri.it (A.R.); emilio.benfenati@marionegri.it (E.B.)

**Keywords:** eye irritation, SAR, SMILES, monte carlo method, semi-correlation, CORAL software

## Abstract

The OECD recognizes that data on a compound’s ability to treat eye irritation are essential for the assessment of new compounds on the market. In silico models are frequently used to provide information when experimental data are lacking. Semi-correlations, as they are called, can be useful to build up categorical models for eye irritation. Semi-correlations are latent regressions that can be used when the endpoint is expressed by two values: 1 for an active molecule and 0 for an inactive molecule. The regression line is based on the descriptor values which serve to distribute the data into four classes: true positive, true negative, false positive, and false negative. These values are applied to calculate the corresponding statistical criterion for assessing the predictive potential of the categorical model. In our model, the descriptor is the sum of what are termed correlation weights. These are defined by optimization using the Monte Carlo method. The target function of the optimization is related to the determination coefficient and the mean absolute error for the training set. Our model gives results that are better than those previously reported for the same endpoint.

## 1. Introduction

The Organization for Economic Cooperation and Development (OECD) recognizes that a compound’s effects on eye irritation are essential in the assessment of new compounds. The OECD has adopted three methods for assessing eye irritation [1,2]. When experimental values are lacking, models for the prediction of the adverse impact of chemicals are essential to modern theoretical chemistry [3,4,5,6], using various representations of the molecular structure [7]. Solving environmental problems by simulation endpoints that are indicators of hazardous or beneficial properties of substances is now a common practice. There are two types of simulation aimed at solving the mentioned problems. There is the regression approach, where endpoints are simulated and expressed as numerical values on a continuous scale or a categorical simulation, with the model taking the form of two values representing active (1) or inactive (0) molecules. Semi-correlation is one of the possible approaches for building a categorical model [8,9]. It should be noted, however, that semi-correlations are latent regression models. The result is a somewhat unusual correlation since the abscissa axis (if we continue the analogy with the usual regression model) displays only two values that can be expressed as 0− to mark an inactive compound, and 1− to mark an active compound. There may be variations such as −1 and +1 to indicate inactive and active compounds, respectively. While the y-axis contains a wide range of values, their informational content boils down to how they are located relative to 0.5 (if the range of observations is 0 and 1), or how they are located relative to 0 (if the range of observations is represented by −1 and +1). For regression models built using the Monte Carlo technique, meaning through self-controlled random processes, a mechanistic interpretation of the model can be presented in probabilistic terms, partially dependent on the distribution of the molecular features extracted from the SMILES. This interpretation is revealed through several runs of procedures to find the so-called correlation weights provided for each of the above-mentioned molecular features of the transmitted SMILES. In this case, the descriptor on which the model is based is a simple sum of these correlation weights calculated by the Monte Carlo method.

Three classes of molecular features can be distinguished during these procedures for calculating correlation weights, repeated several times. The first contains molecular features which, in all the runs of optimization by the Monte Carlo method, receive positive correlation weights. Despite the probabilistic nature of this process, most of these molecular features are extremely likely to be favorable factors for increasing the values of the endpoint studied in the regression. The second class of molecular features under discussion are those that receive a negative correlation weight in each run of the Monte Carlo optimization. Molecular features of this nature will, with fairly significant probability, turn out to be factors that contribute to a decrease in the value of the endpoint in question. Finally, the third class of molecular features extracted from SMILES are those for which both positive and negative correlation weights are encountered during several Monte Carlo optimizations. In principle, by comparing how many times the positive weights and how many times the negative weights are observed, one can assign these molecular features to either the first or second of these classes. For this kind of observation, many runs of the optimization procedure are needed, though in practice the number is limited, and in those circumstances, it is simpler and more logical to classify substances of this third class as molecular features with no clear role in terms of raising or lowering the values of the endpoint in question. 

Then, there are cases when a model (ordinary regression or semi-correlation) is formed on exclusively positive correlation weights (where everyone ends up in the same first class) or exclusively negative correlation weights (all in the second class). In such cases, the only way to understand the contributions of the molecular features in the specified application is to compare the correlation weights and the frequencies of the corresponding molecular features extracted from the SMILES. Since semi-correlations, as already noted, are special cases of regression models, the situations listed here also apply to them. Thus, one can distinguish three classes of molecular features for semi-correlations. Therefore, for an endpoint with only two values (active/inactive), one can apply techniques tested on conventional “traditional” regression models, where the considered endpoint has a certain range of values. 

Here, the semi-correlations are used to develop a categorical model of eye irritation. The CORAL software (http://www.insilico.eu/coral, accessed on 25 November 2023) is a tool for building semi-correlations [8,9]. 

## 2. Materials and Methods 

The data on eye irritation from 5220 chemicals (including 3874 positive and 1346 negative) were taken from the literature [10]. To develop the models, the data were randomly divided into four subsets with approximately equal numbers of chemicals: these are referred to as the active and passive training sets, calibration set, and validation set. The traditional training set is structured in three subsets (active and passive training sets and a calibration set). Special interactions take place in this trio. The active training set is intended for building the initial model, with correlation weights that force the experimental and calculated values of the endpoint to correlate. The passive training set checks how suitable the resulting correlation weights are for the molecules distributed in the passive training set (i.e., absent in the active training set). Last, the calibration set checks for the absence of overtraining (meaning a situation where a very good correlation on the training sets is accompanied by a “fall” in the coefficient of determination for the calibration set). Thus, it is at this stage that the final model is optimized. The validation set is not used in the process of model building and provides the statistics for substances not used in the training sets.

### 2.1. Optimal SMILES-Based Descriptors 

The simplified molecular input-line entry system (SMILES) is a representation of the molecular structure [7]. The optimal descriptor is a sum of correlation weights of SMILES atoms for eye irritation, calculated by the Monte Carlo method described in our previous work [11]:

(1) $$DCW\left(T,N\right)=\sum CW(S_{k})$$

*CW(S_k_)* are the correlation weights (CW) for the attribute of SMILES [7]; *S_k_*, that is, one symbol or a group of symbols which cannot be considered separately) e.g., ‘Cl’, ‘Br’, ‘[N+]’, etc., the physical meaning of SMILES fragments available in the literature [7,8,9] as well as on the Internet (https://www.daylight.com/dayhtml/doc/theory/theory.smiles.html, accessed on 15 October 2023); *T* (=1) is the threshold defining active SMILES atoms (those with a frequency more than *T* in the active training set); *N* is the number of the iterations of the optimization by the Monte Carlo method (*N* = 15). Table 1 contains an example of calculating the optimal SMILES-based descriptor.

### 2.2. Model of Eye Irritation

The model of eye irritation is defined as

(2) $$Y=C_{0}+C_{1}\times DCW(T,N)$$

(3) $$CATEGORY\left(SMILES\right)=\left\{\begin{array}{l} 1\left(active\right),\;ifY\geq 0.5\\ 0\left(inactive\right),\;ifY<0.5 \end{array}\right.$$

### 2.3. Monte Carlo Optimization

Equation (1) needs the numerical data for the *CW*, calculated by the Monte Carlo optimization. Here two target functions (*TF*_0_ and *TF*_1_) for the Monte Carlo optimization are examined:

(4) $${TF}_{0}=r_{AT}+r_{PT}-\left|r_{AT}-r_{PT}\right|\times 0.1$$

(5) $${TF}_{1}={TF}_{0}+{IIC}_{C}\times 0.5$$

$r_{AT}$ and $r_{PT}$ are correlation coefficients between the observed and predicted endpoints for the active and passive training sets, respectively. *IIC_C_* is the index of ideality of correlation [12]. *IIC_C_* is calculated with data on the calibration set as follows:

(6) IICC=rCmin⁡(MAEC,MAEC)+−max⁡(MAEC,MAEC)+−

(7) $$\min\left(x,y\right)=\left\{\begin{array}{c} x,\;if\;x<y\\ y,otherwise \end{array}\right.$$

(8) $$\max\left(x,y\right)=\left\{\begin{array}{c} x,\;if\;x>y\\ y,otherwise \end{array}\right.$$

(9) MAEC−=1N−∑∆k,N is the number of∆k−<0

(10) MAEC+=1N+∑∆k,N is the number of∆k+≥0

(11) $$\Delta_{k}={observed}_{k}-{calculated}_{k}$$

*r_c_* is the correlation coefficient between the observed and calculated values of the endpoint on the calibration set; ‘*c*’ indicates that it belongs to the calibration set. Observed and calculated are the corresponding values of *y* applied to define the corresponding categories (active/inactive).

### 2.4. The System of Self-Consistent Models

The system of self-consistent models [11,13,14] for five random splits into the training (visible) and validation (invisible) sets confirms the good predictive potential of the models. The training set here is divided into active and passive training and calibration sets. Thus, the difference between models reflects the difference in training sets. However, the key attribute of the system of self-consistent models is the unified method for the validation of these models; each *i*-th model has *i*-th validation set. The validation sets are far from identical (Table S1, Supplementary Materials). This supports the statistical fact that we explore multiple conditions, and the results are representative of a set of cases, each obtained by chance, and their overall results should be evaluated jointly.

The measure of self-consistency is the average value and dispersion of the Matthews correlation coefficient (*MCC*) on different validation sets. The corresponding computational experiments are represented by the matrix:

(12) $$\left[\begin{array}{ccc} \left(M_{1}(V_{1}):V_{1}\to {MCCv}_{11}^{}\right) & \cdots & {(M}_{5}(V_{5}):{V\prime }_{1}\to {MCCv}_{51}^{})\\ \vdots & & \vdots \\ {(M}_{1}(V_{1}):{V\prime }_{5}\to {MCCv}_{15}^{}) & \cdots & \left(M_{5}(V_{5}):V_{5}\to {MCCv}_{55}^{}\right) \end{array}\right]$$

$M_{i}$ is an *i*-th model; ${V\prime }_{j}$ is the list of compounds employed as the validation set in the case of the *j*-th split; ${{MCCv}_{ij}^{\;}}_{\;}$ is the Matthews correlation coefficient for the *j*-th validation set if applied to the *i*-th model.

## 3. Results

The models for five random splits are the following:

*Y* = 1.091 + 0.03088 × DCW(1,15) (13)

*Y* = 1.111 + 0.03304 × DCW(1,15) (14)

*Y* = 1.075 + 0.02636 × DCW(1,15) (15)

*Y* = 1.129 + 0.02883 × DCW(1,15) (16)

*Y* = 1.107 + 0.04178 × DCW(1,15) (17)

Table 1 sets out the statistical characteristics of the models for five random splits. One can see that these characteristics are quite similar for all five random splits. The average value of MCC for the validation sets is 0.8891 ± 0.0153. The numbers of the optimized parameters in the Monte Carlo optimization process are 31, 28, 30, 30, and 31, respectively, for splits 1–5.

(18) $$\mathrm{Sens}\;(\mathrm{sensitivity})=\frac{TP}{TP+FN}$$

(19) $$\mathrm{Spec}\;(\mathrm{specificity})=\frac{TN}{TN+FP}$$

(20) $$\mathrm{Acc}\;(\mathrm{accuracy})=\frac{TP+TN}{TP+FP+FN+TN}$$

(21) $$\mathrm{MCC}\;(\mathrm{Mathew}\;\mathrm{correlation}\;\mathrm{coefficient})=\frac{TP\times TN-FP\times FN}{\sqrt{\left(TP+FP)(TP+FN)(TN+FP)(TN+FN\right)}}$$

Table 2 indicates that the evolution of the model enables us to optimize it. The statistics of the active and passive subsets (already very good) are even better for the validation set. The optimized model, as in the calibration set, is expected to provide similar statistics when tested on new substances, as in the case of the validation set. In fact, the values for the calibration and validation sets are quite similar. This provides evidence that the model can be expected to give good results when used for new substances. As described above, the training set is not balanced because there are more toxic substances. In these conditions, the use of the accuracy may provide a biased picture of the model’s performance. It is common that the most represented class is predicted more easily, and indeed in our case we observe that the sensitivity values are higher than the specificity value. To have a better evaluation of the overall performance of the model for the unbalanced datasets, the MCC parameter is more appropriate; MCC ranges from −1 to 1, and good values are those above 0.5.

Table 3 lists the correlation weights of the SMILES atoms for splits 1–5. Many of the SMILES’ attributes are rare, meaning they are present only in a few substances, and their roles in the five splits are different. This implies that these controversial features can hardly be considered as a reliable basis for statistical conclusions. In our case, these attributes are not used in the simulation.

The correlation weights and even their lists are different for the five random splits, but the statistical quality of the models is quite good and close. To explain this, we have to bear in mind that the data set, which is large, with more than 5000 substances, is sufficient to generate several good models, which are not identical, because in our exercise we split these substances randomly. Each model has an adequate, good statistical basis, but depending on the composition of the training set, different features may be extracted.

Table 1 gives an example of the calculation of the property value Y for a simple SMILES.

As noted above, to obtain a basis for determining a mechanistic interpretation, it is necessary to carry out several runs of optimizing the correlation weights of the molecular features extracted from the SMILES. Table 4 gives the results of such tests using splits 1–5. Table 5 indicates that for the system of models under consideration, there are promoters of both “increase/decrease” in the activity. There is a certain analogy in the lists but also differences. Table 6 contains a comparison of the similarities and differences in the mentioned lists.

There are two factors that allow for an evaluation of a molecular feature as a potential promoter of the increase in the activity in question. It should be noted that it is more appropriate to talk not so much about an “increase” as about the “probability of activity” for each specific substance. These factors are the frequency of the occurrence and stability of the correlation weight. For example, bromine (Br) is present in all the lists of promoters of an increase in the likelihood of activity, while nitrogen (n, i.e., nitrogen in an aromatic ring, according to the SMILES nomenclature) is present in all the lists of molecular features reducing the probability of activity for the five splits. The frequency of phosphorus and fluorine is too low to assess them as potential promoters of activity or inactivity (Table 4 and Table 5). We can therefore see whether a certain molecular feature has been selected in all five splits, which is the preferable situation. The value of the coefficient and its consistency is another important feature. If the coefficient is close to 0, its role is lower. If the coefficient is variable, we expect greater uncertainty on its role.

For instance, bromine ‘Br’ contributes to eye irritation, and the coefficients span from 0.11 to 0.42. Silicon and boron are other features consistently contributing to eye irritation with relatively high coefficients. The triple bond (represented by the symbol #) is a feature selected in all the splits, but the coefficients are lower: 0.002 (almost negligible), 0.02 (quite small), and 0.26. In addition, in some splits, there are molecular features with very high coefficients, but these findings are not replicated in other splits. This is the case of charged nitrogen ‘[N+]’, with coefficients of 0.87 and 1.02 in two of the five splits, while it has not been selected in the third. This is probably because of the particular composition of the splits, with certain substances present in the calibration and validation sets.

Similar considerations are held for the molecular features that reduce eye irritation.

A plus denotes the presence of a molecular feature in the list of promoters for an increase or decrease of the “probability of activity”.

The results of our computer experiments set out to identify which molecular fragments of the SMILES have an influence on the likelihood of the substances being able to affect the eyes are presented in Table 5. It can be seen that there are ‘convincing’ supporters for the presence of effects of substances on the eyes such as bromine ‘Br’, silicon ‘[Si]’, nitrogen, ‘N’, and ‘n’. There are also ‘fragments that may have such an influence’ which depends on the distribution of the substances into the training and validation sets, for instance, these are triple bond ‘#’, phosphorus ‘P’, sulfur ‘S’, and some others.

The applicability domain in each split was defined from the statistical defects [15]. The Supplementary Materials section contains this data on split 1 (Table S2). This is not a ‘strict’ way of determining the applicability domain. Outliers for this approach are “suspicious” molecules that have a significant number of rare molecular features.

Table 6 compares the statistical quality of the different categorical models of eye irritation, also considering those published in the recent literature.

The difference between the model-building system considered here and the more generally accepted ones is the use of a structured training set that includes three functionally different groups of compounds. Active and passive learning complement each other, postponing the moment of relearning. The third functional component (a calibration set) should catch the moment when overtraining starts. However, overtraining may not occur at all due to the use of the index of ideality of correlation. In other words, the feasibility of using the index of ideality of correlation to improve the statistical quality of the different models for the external validation sets is once again demonstrated for various substances and endpoints [12,16,17].

## 4. Discussion

The disadvantage of the scheme considered here (system of self-consistent models) is the need to compare a sufficiently large amount of digital data, to divide all the available substances into four sets. Furthermore, only considering the various groups of splits into training and validation sets can give an idea of the genuine predictive potential of a particular approach. The approach used here to generate the splits is through a random process. If we consider a random process as a sequence of changes in random variables, then, in the case under consideration, the random variables are the determination coefficients and their standard deviations. We get two levels of random processes. The first level of change in the specified quantities for one random process is building a model for a fixed division into the training and validation sets. The second level is the consideration of a certain group of random processes for different splits into the training and validation sets. The second level is designed to answer the following questions. Question 1: Is it possible to build up a model with our approach to selected parameters? Question 2: How reproducible are the statistical characteristics of the model in the chosen parameterization for various distributions into the training and validation sets? Question 3: If the statistical characteristics are reproducible, what is their variance?

By knowing the answers to these questions, one can assess how reliable the model is. The self-consistency of the models does not signify their identity. Each model is different, as we discussed above, but each model may be appropriate. Even the proportions in the distribution of available data into the active/passive training, calibration, and validation sets are not subject to any restrictions. However, the mentioned proportions, according to many computational experiments, are the best when using equivalent numbers of compounds for each of the four mentioned sets.

The main methodological novelty applied in this study is the improved exploitation of the statistical parameters governing the modelling task. It is possible by chance to get an apparently good model, but unfortunately the results will not be replicated when applying the model to new substances. The algorithms that we implemented in the past and then applied here are useful to reduce the risk of overfitting the models. The approach at the basis for this, as explained above, relates to the algorithms of the index of ideality of correlation and the system of self-consistent models. This approach necessitates splitting into different subsets, which means added complexity, as discussed above. However, at the same time, this provides the opportunity to optimize the parameters of the final model, such as the coefficients, addressing the calibration sets. This approach is rewarding in terms of the performance on the validation set, as documented above.

Protecting animals from cruelty is one of the main motivations for the development of QSAR in general, as well as using QSAR to estimate eye irritation [18]. To this end, the development of binary models that give a forecast in the form of “active”—“inactive” is encouraged. There are developments aimed at building multivariate categorical models of eye irritation obtained through decision trees for individual substances [18], as well as for mixtures [19]. Any models for eye irritation are very unstable in cases of significant structural variations in molecules [6]. To overcome these difficulties, it is necessary, on the one hand, to improve (expand) the training databases and, on the other hand, to improve methods for validation of the predictive potential of the models [20,21]. Taking these circumstances into account, one of the most representative databases was selected to test the considered approach, and the self-consistency of the models was studied to search for ways to improve the criteria of the predictive potential of the models. The simplicity is on the verge of primitiveness (since only the simplest features of molecular structures are involved), which is the basis for the development of models of eye irritation. This, combined with the usual statistical assessment on the principle of comparing average values and their variances, perhaps is the main cause of the good statistical quality of the models (note that complication almost always leads to overtraining). The involvement of the correlation ideality index somewhat complicates the stochastic Monte Carlo optimization process. However, this complication is justified here and in other works where the mentioned index was used [11,12,13,14,17].

We note that this model starts with substances classified into two classes: active or not. Thus, there is no information about the potency of the effect. In other cases, for instance, skin sensitization and the granularity of the effects have been introduced. For eye irritation specifically, it would be convenient in the future to distinguish between the effects with different severities, considering chemical burns (irritation versus corrosion).

## 5. Conclusions

Building models using the structurization of the compounds selected for the training set into three functionally different subsets can effectively improve the predictive potential of the categorical models for eye irritation using semi-correlations. The unusual impact of the index of ideality of correlation on the simulation process allows for an improvement in the statistical quality of the model for the external validation set based on the good results of the calibration set. This improves the predictive potential of the categorical models. Using this methodology, we obtained good models for eye irritation. The model is relatively simple since it only requires the SMILES structure of the substances without calculating the molecular descriptors. The model’s interpretation of the molecular parameters is simple as well since it points out which atom or simple molecular feature indicates the increase or decrease of the activity. However, it should be noted that these recommendations are of a qualitative (non-quantitative) nature.

## Figures and Tables

**Table 1:** An example of model calculation CAS = 503-17-3; SMILES = CC#CC.

*S_k_*	*CW(S_k_)*	The Frequency in Active Training Set	The Frequency in Passive Training Set	The Frequency in Calibration Set
C	−0.8947	1199	1177	1130
C	−0.8947	1199	1177	1130
# (triple bond)	2.5018	67	70	77
C	−0.8947	1199	1177	1130
C	−0.8947	1199	1177	1130
$\sum CW(S_{k})$	−1.0769			

*Y* = 1.091 + 0.03088 × (−1.0769) = 1.057 (active because *Y* is larger than 0.5).

**Table 2:** The statistical characteristics of the models for eye irritation for five random splits.

Split	Set *	Sens	Spec	Acc	MCC	TN	TP	FP	FN	All
1	A	0.9303	0.5459	0.8166	0.5332	868	214	178	65	1325
	P	0.9193	0.5407	0.7974	0.5117	809	226	192	71	1298
	C	0.9856	0.8842	0.9654	0.8897	1026	229	30	15	1300
	V	0.9804	0.8917	0.9614	0.8839	1000	247	30	20	1297
2	A	0.9247	0.5639	0.8122	0.5397	847	234	181	69	1331
	P	0.9461	0.6250	0.8480	0.6267	843	245	147	48	1283
	C	0.9941	0.9163	0.9781	0.9321	1008	241	22	6	1277
	V	0.9858	0.9130	0.9707	0.9099	1038	252	24	15	1329
3	A	0.9322	0.5427	0.8140	0.5342	852	216	182	62	1312
	P	0.9381	0.5661	0.8285	0.5639	849	214	164	56	1283
	C	0.9800	0.8780	0.9568	0.8753	980	259	36	20	1295
	V	0.9867	0.8545	0.9594	0.8734	1041	235	40	14	1330
4	A	0.8990	0.5633	0.7860	0.5002	783	249	193	88	1313
	P	0.9237	0.5807	0.8093	0.5533	823	259	187	68	1337
	C	0.9953	0.9558	0.9884	0.9595	1060	216	10	5	1291
	V	0.9943	0.9310	0.9828	0.9414	1041	216	16	6	1279
5	A	0.9147	0.5523	0.7960	0.5145	826	243	197	77	1343
	P	0.9198	0.5381	0.7894	0.5109	791	240	206	69	1306
	C	0.9922	0.9826	0.9905	0.9683	1020	226	4	8	1258
	V	0.9972	0.9826	0.9947	0.9815	1080	226	4	3	1313

* A = active training set; P = passive training set; C = calibration set; V = validation set; TN = true negative; TP = true positive; FN = false negative; FP = false positive; All is the number of compounds in a set.

**Table 3:** The correlation weights for SMILES atoms that are used to calculate *Y* in the case of splits 1–5.

*S_k_*	*CW(S_k_)*	The Frequency in Active Training Set	The Frequency in Passive Training Set	The Frequency in Calibration Set	*S_k_* is Active
**Split 1**					
#	−0.0018	67	70	77	TRUE
(	0.4051	1136	1122	1058	TRUE
/	0.0	0	1	0	FALSE
1	0.1432	1080	1066	1058	TRUE
2	−0.0537	526	529	452	TRUE
3	0.2177	244	262	226	TRUE
4	−0.1914	112	123	108	TRUE
5	0.4467	33	41	34	TRUE
6	0.3766	7	15	12	TRUE
7	0.0835	5	6	4	TRUE
8	0.4559	2	3	3	TRUE
9	0.0	0	1	1	FALSE
=	0.4918	827	852	791	TRUE
B	−0.4305	4	2	10	TRUE
C	0.3553	1199	1177	1130	TRUE
F	0.4496	104	91	118	TRUE
Br	0.3882	70	56	115	TRUE
I	0.3796	24	34	32	TRUE
Cl	0.3870	185	172	195	TRUE
N	0.2175	601	597	514	TRUE
O	−0.2029	1010	995	939	TRUE
P	−0.1273	10	24	9	TRUE
S	0.1072	139	137	140	TRUE
[123I]	0.0	0	1	0	FALSE
\	0.0	0	1	0	FALSE
[C−]	0.0	0	0	1	FALSE
[Ge]	0.0	1	0	1	FALSE
[N+]	0.4829	66	76	98	TRUE
[O−]	−0.2889	70	76	99	TRUE
[Se]	0.0	0	0	1	FALSE
[Si]	0.4429	5	8	12	TRUE
[Sn]	−0.1457	3	1	1	TRUE
[n+]	0.1103	5	1	2	TRUE
[nH]	−0.2015	51	39	39	TRUE
c	0.1066	921	909	940	TRUE
n	−0.1502	213	214	202	TRUE
o	−0.3202	21	17	23	TRUE
s	−0.3532	30	26	43	TRUE
**Split 2**					
#	−0.0156	69	60	80	TRUE
(	−0.4088	1152	1118	1036	TRUE
/	0.0	0	0	1	FALSE
1	−0.4359	1107	1061	991	TRUE
2	−0.4951	525	501	437	TRUE
3	0.0739	254	245	227	TRUE
4	0.1088	117	99	118	TRUE
5	0.3121	37	33	27	TRUE
6	−0.3768	9	13	6	TRUE
7	0.1469	4	5	2	TRUE
8	0.0	0	3	1	FALSE
9	0.0	0	1	0	FALSE
=	0.3761	840	837	801	TRUE
B	−0.1819	6	4	7	TRUE
C	0.0391	1205	1164	1112	TRUE
F	0.3489	90	104	127	TRUE
Br	0.4237	69	56	97	TRUE
I	−0.3217	25	46	25	TRUE
Cl	0.3727	183	180	184	TRUE
N	−0.2051	619	569	521	TRUE
O	−0.3873	1012	997	941	TRUE
P	−0.2234	12	19	6	TRUE
S	0.4145	157	125	123	TRUE
[123I]	0.0	0	0	1	FALSE
[18F]	0.0	0	0	1	FALSE
\	0.0	0	0	1	FALSE
[C−]	0.0	0	1	0	FALSE
[Ge]	0.0	0	0	2	FALSE
[N+]	−0.1110	82	81	75	TRUE
[O−]	0.3385	82	84	78	TRUE
[Si]	0.2676	8	9	15	TRUE
[Sn]	0.0	1	2	0	FALSE
[n+]	0.0	0	5	4	FALSE
[nH]	−0.0460	41	39	40	TRUE
c	−0.4981	953	895	868	TRUE
n	0.1179	220	191	176	TRUE
o	0.2430	21	25	15	TRUE
s	0.1486	36	23	31	TRUE
**Split 3**					
#	0.2631	67	60	77	TRUE
(	−0.3882	1109	1092	1105	TRUE
/	0.0	1	0	2	FALSE
1	−0.4166	1072	1048	1042	TRUE
2	−0.2890	511	509	471	TRUE
3	0.1485	245	254	252	TRUE
4	0.1888	115	118	117	TRUE
5	−0.3245	35	35	38	TRUE
6	0.3899	13	14	10	TRUE
7	0.3989	6	6	7	TRUE
8	0.0	1	4	4	FALSE
9	0.0	1	3	0	FALSE
=	−0.3768	838	825	824	TRUE
B	0.1439	4	2	7	TRUE
C	−0.4266	1179	1140	1145	TRUE
F	−0.1818	98	104	113	TRUE
Br	0.1097	68	58	96	TRUE
I	−0.4305	30	33	34	TRUE
Cl	−0.0516	180	185	189	TRUE
N	0.3385	605	544	545	TRUE
O	−0.0065	988	979	975	TRUE
P	−0.3230	13	16	11	TRUE
S	0.3219	141	131	126	TRUE
[123I]	0.0	0	1	0	FALSE
[18F]	0.0	0	1	0	FALSE
\	0.0	1	0	2	FALSE
[Ge]	0.0	1	0	1	FALSE
[N+]	0.1284	76	75	82	TRUE
[O−]	−0.4385	78	76	84	TRUE
[Si]	0.4327	13	7	14	TRUE
[Sn]	0.0	1	2	3	FALSE
[V]	0.0	1	0	0	FALSE
[n+]	−0.0209	2	1	3	TRUE
[nH]	−0.1779	34	44	44	TRUE
c	0.4635	918	881	932	TRUE
n	−0.2132	210	198	200	TRUE
o	−0.3341	29	22	17	TRUE
s	−0.4718	35	29	39	TRUE
**Split 4**					
#	0.2930	58	59	81	TRUE
(	−0.4336	1157	1151	1068	TRUE
/	0.0	0	1	0	FALSE
1	0.1723	1102	1128	991	TRUE
2	−0.3311	566	559	421	TRUE
3	0.0101	286	258	214	TRUE
4	−0.1253	132	114	103	TRUE
5	0.0920	41	38	27	TRUE
6	−0.1034	16	12	8	TRUE
7	0.4945	6	7	5	TRUE
8	0.0289	4	3	2	TRUE
9	0.0	1	1	2	FALSE
=	0.4146	879	868	789	TRUE
B	−0.0762	2	2	11	TRUE
C	−0.0174	1180	1212	1129	TRUE
F	0.3847	88	104	124	TRUE
Br	0.4093	57	60	104	TRUE
I	−0.2812	28	28	31	TRUE
Cl	−0.4663	191	193	187	TRUE
N	0.3680	650	648	467	TRUE
O	0.2090	1022	1043	944	TRUE
P	−0.1516	19	11	13	TRUE
S	0.4176	146	155	111	TRUE
[123I]	0.0	0	0	1	FALSE
[18F]	0.0	0	1	0	FALSE
\	0.0	0	1	0	FALSE
[C−]	0.0	0	0	1	FALSE
[Ge]	0.0	0	1	0	FALSE
[N+]	0.1838	74	69	100	TRUE
[O−]	−0.1966	75	71	105	TRUE
[Se]	0.0	0	0	1	FALSE
[Si]	0.0827	6	8	19	TRUE
[Sn]	0.2362	3	1	2	TRUE
[V]	0.0	1	0	0	FALSE
[n+]	0.0	1	3	7	FALSE
[nH]	0.3438	42	43	37	TRUE
c	0.0892	948	962	869	TRUE
n	0.3388	198	207	198	TRUE
o	−0.2745	27	19	8	TRUE
s	−0.2667	30	36	34	TRUE
**Split 5**					
#	−0.1927	58	68	90	TRUE
(	0.1698	1169	1136	1036	TRUE
/	0.0	1	1	0	FALSE
1	−0.1953	1111	1084	1005	TRUE
2	0.0434	571	555	417	TRUE
3	0.4368	268	270	207	TRUE
4	0.4982	123	108	117	TRUE
5	0.4949	35	31	36	TRUE
6	−0.2375	10	10	9	TRUE
7	0.4610	6	4	4	TRUE
8	0.1974	3	2	3	TRUE
9	0.0551	2	0	1	TRUE
=	−0.2514	861	864	779	TRUE
B	−0.3615	3	3	7	TRUE
C	−0.0744	1223	1177	1094	TRUE
F	−0.0703	92	107	110	TRUE
Br	0.4524	49	67	101	TRUE
I	−0.3837	32	27	25	TRUE
Cl	0.1357	206	182	182	TRUE
N	−0.2709	646	642	489	TRUE
O	−0.0999	1038	1006	926	TRUE
P	−0.3508	9	22	10	TRUE
S	−0.0258	144	146	126	TRUE
[123I]	0.0	0	1	0	FALSE
[18F]	0.0	0	1	0	FALSE
\	0.0	1	1	0	FALSE
[C−]	0.0	1	0	0	FALSE
[Ge]	−0.2719	2	0	0	TRUE
[N+]	0.1633	62	78	113	TRUE
[O−]	−0.4126	62	81	118	TRUE
[Se]	0.0	0	1	0	FALSE
[Si]	−0.4059	11	10	11	TRUE
[Sn]	0.0	1	0	4	FALSE
[V]	0.0	1	0	0	FALSE
[n+]	0.0	1	3	7	FALSE
[nH]	0.0709	34	34	50	TRUE
c	−0.2732	942	939	891	TRUE
n	−0.1253	221	239	179	TRUE
o	0.0797	21	19	20	TRUE
s	−0.1934	34	38	25	TRUE

**Table 4:** The roles of the different molecular features to provide the basis for the mechanistic interpretation in terms of the three classes of molecular features.

No.	Molecular Feature	CWs Probe 1	CWs Probe 2	CWs Probe 3	CWs Probe 4	CWs Probe 5	NA *	NP	NC	d
	Split 1 class 1									
1	Br	3.2723	1.3463	1.7811	4.0953	3.1221	70	56	115	0.0004
2	[O−]	1.7365	0.6665	0.5790	2.1466	0.7586	70	76	99	0.0002
3	#	3.8341	1.9513	1.2741	0.8575	4.4929	67	70	77	0.0001
4	7	3.2718	1.5793	1.7186	2.7828	1.6371	5	6	4	0.0002
5	[Si]	3.4661	3.0762	2.1314	1.2030	5.8817	5	8	12	0.0004
6	[n+]	6.8173	3.3466	4.4202	4.5583	8.5185	5	1	2	0.0008
7	B	4.7752	1.3899	2.2479	3.1393	2.0450	4	2	10	0.0008
8	[Sn]	9.0484	7.1590	8.8854	8.6893	12.0496	3	1	1	0.0006
	Split 1 class 2									
1	C	−0.6605	−0.3548	−0.5480	−0.8370	−0.5159	1199	1177	1130	0.0000
2	(	−0.3212	−0.4346	−0.0967	−0.0620	−0.9159	1136	1122	1058	0.0000
3	1	−1.6947	−0.3518	−0.2589	−0.8020	−1.4060	1080	1066	1058	0.0000
4	N	−2.7232	−1.0325	−2.1581	−1.6193	−3.0509	601	597	514	0.0001
5	2	−2.7013	−1.9285	−1.4587	−2.0087	−3.3464	526	529	452	0.0001
6	3	−1.7331	−0.6932	−1.1344	−2.0032	−2.5845	244	262	226	0.0001
7	n	−1.4273	−1.2580	−0.5318	−1.2792	−1.4411	213	214	202	0.0000
8	Cl	−0.8715	−0.4565	−0.6725	−1.6398	−0.1631	185	172	195	0.0001
9	S	−2.5577	−0.9998	−1.7481	−1.2768	−1.8630	139	137	140	0.0000
10	s	−4.8609	−2.5041	−0.2666	−0.7957	−2.6770	30	26	43	0.0003
11	o	−4.3739	−1.2544	−1.9536	−2.0792	−4.2302	21	17	23	0.0002
	Split 2 Class 1									
1	F	1.4318	0.4211	0.6747	0.6329	0.7588	90	104	127	0.0002
2	[N+]	0.8732	0.6956	1.7182	0.1880	1.2008	82	81	75	0.0000
3	[O−]	0.5347	0.1252	2.4039	0.9312	0.5493	82	84	78	0.0000
4	#	2.8296	2.5711	3.7341	2.4279	2.4808	69	60	80	0.0002
5	Br	3.1784	2.1593	2.2501	1.5241	1.3780	69	56	97	0.0003
6	P	3.2709	2.9828	2.9690	1.3972	2.1455	12	19	6	0.0005
7	[Si]	2.2949	4.3398	3.4141	1.9805	2.3602	8	9	15	0.0004
8	B	2.9435	2.3674	3.1590	1.8198	1.4780	6	4	7	0.0003
9	7	4.6291	3.5557	3.3704	1.7851	2.7409	4	5	2	0.0004
	Split 2 Class 2									
1	C	−0.4048	−0.7736	−0.3752	−0.3051	−0.3977	1205	1164	1112	0.0000
2	(	−0.6309	−0.1135	−0.3682	−0.1438	−0.2676	1152	1118	1036	0.0000
3	N	−2.2544	−2.4720	−2.1661	−1.1186	−1.4333	619	569	521	0.0001
4	2	−3.5296	−2.3461	−3.1806	−1.5277	−1.8080	525	501	437	0.0001
5	3	−2.1478	−1.6436	−1.7882	−1.2203	−1.0766	254	245	227	0.0000
6	n	−0.6723	−1.2947	−0.7581	−0.6257	−0.8781	220	191	176	0.0001
7	Cl	−2.0880	−1.0896	−1.1427	−1.1099	−0.7095	183	180	184	0.0000
8	S	−1.0411	−1.2982	−0.4788	−0.5819	−0.6556	157	125	123	0.0001
9	4	−0.3482	−0.2583	−1.2454	−0.3755	−0.6052	117	99	118	0.0001
10	[nH]	−1.1206	−1.8879	−1.5457	−0.9128	−0.9076	41	39	40	0.0000
11	s	−3.4152	−1.4488	−2.6476	−1.7933	−0.6432	36	23	31	0.0002
12	o	−2.1937	−4.0809	−0.1840	−0.2794	−0.7211	21	25	15	0.0003
	Split 3 Class 1									
1	[N+]	1.0164	0.9943	1.3058	0.2875	0.9219	76	75	82	0.0000
2	Br	1.1498	1.3565	0.9857	2.3486	3.1166	68	58	96	0.0003
3	#	1.3830	1.6466	1.0097	3.3559	3.7289	67	60	77	0.0001
4	6	1.5681	0.4792	1.2337	1.3321	0.3680	13	14	10	0.0002
5	P	1.7308	1.4137	1.7629	2.1308	1.5255	13	16	11	0.0002
6	[Si]	0.5803	0.9440	1.1057	3.2908	3.0645	13	7	14	0.0003
7	7	0.7650	0.9308	0.1770	1.6979	2.0971	6	6	7	0.0001
8	[n+]	0.9517	1.4603	0.8602	0.9493	3.3557	2	1	3	0.0005
	Split 3 Class 2									
1	C	−0.4496	−0.2875	−0.4625	−0.2968	−0.3722	1179	1140	1145	0.0000
2	(	−0.0166	−0.1634	−0.1578	−0.3955	−0.5715	1109	1092	1105	0.0000
3	N	−1.8463	−1.1613	−1.4371	−1.4782	−1.8784	605	544	545	0.0000
4	2	−1.7822	−1.1166	−1.1200	−2.4887	−2.7523	511	509	471	0.0000
5	3	−0.8123	−0.7781	−1.0673	−1.4683	−2.2162	245	254	252	0.0000
6	n	−0.8070	−0.4791	−0.7562	−0.4099	−0.4217	210	198	200	0.0000
7	Cl	−0.6271	−0.1657	−1.2458	−0.4402	−0.8417	180	185	189	0.0000
8	S	−0.8774	−0.9040	−0.5881	−0.8678	−1.3786	141	131	126	0.0001
9	[nH]	−0.5969	−0.6678	−1.4561	−0.8938	−1.4308	34	44	44	0.0001
10	I	−1.3131	−0.0016	−1.9577	−1.2033	−1.6649	30	33	34	0.0001
	Split 4 Class 1									
1	#	3.0592	0.4275	1.7748	2.8090	2.3299	58	59	81	0.0002
2	Br	2.5508	3.0172	2.7507	1.7440	1.2147	57	60	104	0.0003
3	5	0.0405	1.6685	0.4280	0.6631	0.9388	41	38	27	0.0002
4	P	0.9044	2.9989	2.5214	1.5331	1.1623	19	11	13	0.0003
5	6	0.6599	0.5148	1.3528	0.3958	0.6901	16	12	8	0.0003
6	[Si]	2.2987	1.5032	2.2809	3.0191	1.7738	6	8	19	0.0006
7	8	1.2541	2.2047	3.6143	1.6287	0.9238	4	3	2	0.0003
8	[Sn]	4.6790	10.8882	7.4711	6.7199	4.0803	3	1	2	0.0005
	Split 4 Class 2									
1	C	−0.4159	−0.8717	−0.7904	−0.4031	−0.3871	1180	1212	1129	0.0000
2	(	−0.0114	−0.2922	−0.2456	−0.1328	−0.0042	1157	1151	1068	0.0000
3	O	−0.6308	−0.2245	−0.3892	−0.0529	−0.3235	1022	1043	944	0.0000
4	c	−0.0389	−0.1040	−0.4182	−0.1399	−0.2246	948	962	869	0.0000
5	N	−1.2370	−2.7788	−2.5813	−1.2900	−0.7878	650	648	467	0.0002
6	2	−2.1037	−3.3300	−3.0946	−1.7167	−1.0090	566	559	421	0.0001
7	3	−1.9136	−2.5553	−1.4174	−1.5132	−0.8398	286	258	214	0.0001
8	n	−0.9712	−1.8482	−2.3349	−0.7493	−0.7850	198	207	198	0.0000
9	Cl	−1.1802	−1.5431	−1.5226	−0.8968	−0.5393	191	193	187	0.0000
10	S	−0.9590	−1.8499	−1.1759	−0.8525	−0.7025	146	155	111	0.0001
11	[nH]	−1.3662	−1.0136	−1.0906	−0.8572	−0.4932	42	43	37	0.0001
12	I	−2.4835	−4.0180	−2.4387	−2.0084	−1.2164	28	28	31	0.0001
	Split 5 Class 1									
1	F	1.1998	1.4944	0.2246	0.5633	1.2588	92	107	110	0.0001
2	Br	4.0569	3.8501	1.1048	2.3848	3.4291	49	67	101	0.0004
3	[Si]	4.0676	4.8714	1.0975	3.1740	2.9609	11	10	11	0.0001
4	6	1.0129	1.5077	1.8156	0.7851	1.7448	10	10	9	0.0000
5	P	2.5960	1.5276	0.9044	1.5002	2.1112	9	22	10	0.0005
6	7	1.7390	3.6003	0.9580	1.2172	3.0213	6	4	4	0.0002
7	9	5.0624	2.5242	2.8513	4.9072	3.0430	2	0	1	0.0010
8	[Ge]	9.7682	6.3697	3.5542	5.5588	4.3939	2	0	0	1.0000
	Split 5 Class 2									
1	C	−0.4755	−0.4418	−0.3983	−0.6964	−0.5647	1223	1177	1094	0.0000
2	(	−0.6192	−0.5926	−0.1694	−0.2048	−0.4470	1169	1136	1036	0.0000
3	1	−2.5197	−0.1753	−0.9520	−0.2585	−1.7510	1111	1084	1005	0.0000
4	N	−2.5202	−1.5783	−1.4051	−2.0748	−2.0134	646	642	489	0.0001
5	2	−3.3695	−2.4920	−1.0677	−2.1892	−2.7241	571	555	417	0.0001
6	3	−1.8686	−2.3009	−1.0730	−1.1007	−1.7777	268	270	207	0.0001
7	n	−1.3633	−0.5961	−0.6129	−1.0973	−1.1798	221	239	179	0.0001
8	[nH]	−2.6989	−3.0141	−1.4830	−0.6964	−2.4150	34	34	50	0.0002
9	o	−2.4007	−2.4849	−1.9940	−3.0845	−2.4639	21	19	20	0.0000

* NA, NP, and NC are the frequencies of a molecular feature in active training, passive training, and calibration set, respectively.

**Table 5:** Frequency of occurrence of different molecular features in lists of promoters of increase or decrease in probability of the activity observed for five random splits.

	Molecular Feature	Split 1	Split 2	Split 3	Split 4	Split 5
Class 1	Br	+	+	+	+	+
	[O−]	+	+			
	#	+	+	+	+	
	7	+	+	+		+
	[Si]	+	+	+	+	+
	[n+]	+		+		
	B	+	+	+		
	[Sn]	+			+	
	P		+	+	+	+
	F		+			+
	[N+]		+	+		
Class 2	C	+	+	+	+	+
	(	+	+	+	+	+
	1	+				+
	N	+	+	+	+	+
	2	+	+	+	+	+
	3	+	+	+	+	+
	n	+	+	+	+	+
	Cl	+	+	+	+	
	S	+	+	+	+	
	s	+	+			
	o	+	+			+
	4		+			
	[nH]		+	+	+	+
	I			+	+	

**Table 6:** Statistical characteristics of models of eye irritation for the external validation set.

Method	Accuracy	Sensitivity	Specificity	References
Support vector machine	0.938	0.967	0.856	[10]
Artificial neural networks	0.929	0.960	0.845	[10]
Average values on three splits	0.964	0.984	0.886	In this work

## Data Availability

The original data presented in the study are included in the article/Supplementary Material; further inquiries can be directed to the corresponding author.

## Supplementary Materials

The following supporting information can be downloaded at: https://www.mdpi.com/article/10.3390/toxics11120993/s1, Table S1: Confirmation of differences for used splits; Table S2: Technical data of model for split #1.
